# Phytochemical, Antimicrobial and Antiprotozoal Evaluation of *Garcinia Mangostana* Pericarp and α-Mangostin, Its Major Xanthone Derivative

**DOI:** 10.3390/molecules180910599

**Published:** 2013-09-02

**Authors:** Shaza M. Al-Massarani, Ali A. El Gamal, Nawal M. Al-Musayeib, Ramzi A. Mothana, Omer A. Basudan, Adnan J. Al-Rehaily, Mohamed Farag, Mahmoud H. Assaf, KamalEldin H. El Tahir, Louis Maes

**Affiliations:** 1 Department of Pharmacognosy, College of Pharmacy, King Saud University, P.O. Box 2457, Riyadh 11451, Saudi Arabia; E-Mails: salmassarani@ksu.edu.sa (S.M.A.-M.); nalmusayeib@ksu.edu.sa (N.M.A.-M.); r_mothana@yahoo.com (R.A.M.); omer_basodan@yahoo.com (O.A.B.); ajalreha@ksu.edu.sa (A.J.A.-R.); dr.farag@gmail.com (M.F.); 2 Department of Pharmacognosy, Faculty of Pharmacy, Mansoura University, El-Mansoura 35516, Egypt; 3 Department of Pharmacognosy, Faculty of Pharmacy, Assiut University, Assiut P.O. Box 71515, Egypt; E-Mail: mhassaf3000@yahoo.com; 4 Department of Pharmacology, College of Pharmacy, King Saud University, P.O. Box 2457, Riyadh 11451, Saudi Arabia; E-Mail: ktahir@ksu.edu.sa; 5 Laboratory for Microbiology, Parasitology and Hygiene (LMPH), Faculty of Pharmaceutical, Biomedical and Veterinary Sciences, Antwerp University, Universiteitsplein 1, B-2610 Wilrijk-Antwerp, Belgium; E-Mail: louis.maes@ua.ac.be

**Keywords:** *Garcinia mangostana*, α-mangostin, *in vitro*, antiplasmodial, antileishmanial, antitrypanosomal

## Abstract

Five xanthone derivatives and one flavanol were isolated from the dichloromethane extract of *Garcinia mangostana.* Dichloromethane, ethyl acetate extract and the major xanthone (α-mangostin) were evaluated *in vitro* against erythrocytic schizonts of *Plasmodium falciparum*, intracellular amastigotes of *Leishmania infantum* and *Trypanosoma cruzi* and free trypomastigotes of *T. brucei.* The major constituent α-mangostin was also checked for antimicrobial potential against *Candida albicans*, *Escherichia coli*, *Pseudomonas aeruginosa*, *Bacillius subtilis*, *Staphylococcus aureus*, *Mycobacterium smegmatis*, *M. cheleneoi*, *M. xenopi* and *M. intracellulare*. Activity against *P.* *falciparum* (IC_50_ 2.7 μg/mL) and *T. brucei* (IC_50_ 0.5 μg/mL) were observed for the dichloromethane extract, however, with only moderate selectivity was seen based on a parallel cytotoxicity evaluation on MRC-5 cells (IC_50_ 9.4 μg/mL). The ethyl acetate extract was inactive (IC_50_ > 30 µg/mL). The major constituent α-mangostin showed rather high cytotoxicity (IC_50_ 7.5 µM) and a broad but non-selective antiprotozoal and antimicrobial activity profile. This *in vitro* study endorses that the antiprotozoal and antimicrobial potential of prenylated xanthones is non-conclusive in view of the low level of selectivity.

## 1. Introduction

The genus *Garcinia* (Guttiferae, syn. Clusiaceae) contains well-known fruit trees with about 35 genera and up to 800 species of which the fruits of many are edible and serve as a substitute for tamarinds in curries [1]. *Garcinia mangostana* Linn., known as mangosteen, is cultivated in the tropical rainforest of Southeast Asian nations like Indonesia, Malaysia, Sri Lanka, Philippines and Thailand where traditional medicine uses the pericarp for the treatment of abdominal pain, diarrhea, cystitis, eczema, dysentery, wound suppuration and chronic ulcers [2,3]. *In vitro* and *in vivo* laboratory studies have demonstrated that extracts of *G. mangostana* have very diverse pharmacological activities including anti-inflammatory, cytotoxic, antioxidant, antitumoral, immunomodulatory, neuroprotective, anti-allergic, antibacterial and antiviral properties [4,5,6,7]. Phytochemical investigation of the pericarp of *G.* *mangostana* revealed the presence of prenylated xanthones, benzophenones, bioflavonoids and triterpenes [8,9,10]. Over 68 xanthone-type constituents were reported [11], of which the prenylated cage-type is particularly encouraging for further biological and chemical studies. The most studied xanthones are the α-, β-, and γ-mangostins, garcinone E, 8-deoxygartanin and gartanin [7,12].

The present study evaluated the *in vitro* antileishmanial, antiplasmodial and antitrypanosomal potential of the dichloromethane and ethyl acetate extracts of *G.* *mangostana*, as well as the isolation and characterization of its xanthone constituents.

## 2. Results and Discussion

### 2.1. Phytochemical Study

**Figure 1:**
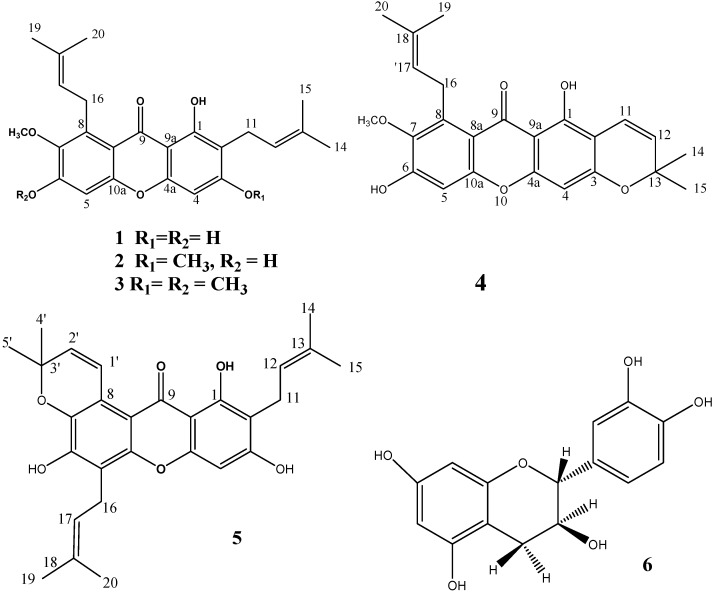
Structures of compounds **1**–**6**.

Chromatographic separation and purification of the dichloromethane extract of *G.* *mangostana* pericarp produced the compounds **1**–**6** (Figure 1). NMR-data (Table 1 and Table 2) and comparison with reported data led to the identification of α-mangostin (**1**) [13], β-mangostin (**2**) [14], 1-hydroxy-3,6,7-trimethoxy-2,8-bis (3-methylbut-2-enyl) xanthone (**3**) [15], 9-hydroxycalabaxanthone (**4**) [16,17], tovophyllin A (**5**) [18,19] and catechin (**6**) [20]. α-Mangostin was the major compound isolated from these series, enabling *in vitro* antiprotozoal and antimicrobial evaluation.

**Table 1:** ^1^H-NMR (500 MHz) spectral data of xanthones **1**–**5**.

Position	Compound 1 ^1^	Compound 2 ^2^	Compound 3 ^2^	Compound 4 ^2^	Compound 5 ^2^
**1**	13.72, *s*	13.42, *s*	13.44, *s*	13.72, *s*	13.79, *s*
**4**	6.28, s	6.24, s	6.30, *s*	6.26, *s*	6.37, *s*
**5**	6.80, *s*	6.74, *s*	6.75, *s*	6.85, *s*	−
**11**	3.35, *d* (*J* = 7.3 Hz)	3.37, *d* (*J* = 7.2 Hz)	3.36, *d* (*J* = 7.1 Hz)	6.74, *d* (*J* = 10.0 Hz)	3.48, *d* (*J* = 6.0 Hz)
**12**	5.17, *t* (*J* = 7.3 Hz)	5.17, *t* (*J* = 7.2 Hz)	5.26, *t* (*J* = 7.1 Hz)	5.58, *d* (*J* = 10.0 Hz)	5.31, *t* (*J* = 7.0 Hz)
**14**	1.77, *s*	1.75, *s*	1.70, *s*	1.27, *s*	1.79, *s*
**15**	1.63, *s*	1.62, *s*	1.71, *s*	1.28, *s*	1.71, *s*
**16**	4.04, *d* (*J* = 7.0 Hz)	4.09, *d* (*J* = 7.2 Hz)	4.15, *d* (*J* = 7.2, Hz)	4.10, *d* (*J* = 7.0 Hz)	3.59, *d* (*J* = 6.0 Hz)
**17**	5.17, *t* (*J* = 7.3 Hz)	5.18, *t* (*J* = 7.2 Hz)	5.26, *t* (*J* = 7.2 Hz)	5.27, *t* (*J* = 7.3 Hz)	5.31, *t* (*J* = 7.0 Hz)
**19**	1.71, *s*	1.61, *s*	1.70, *s*	1.71, *s*	1.87, *s*
**20**	1.73, *s*	1.72, *s*	1.82, *s*	1.82, *s*	1.89, *s*
**3-OMe**		3.82, *s*	3.91, *s*		
**6-OMe**			3.97, *s*		
**7-OMe**	3.71, *s*	3.80, *s*	3.82, *s*	3.83, *s*	
**1'**					8.00, *d* (*J* = 10.0 Hz)
**2'**					5.79, *d* (*J* = 10.0 Hz)
**4'**					1.51, *s*
**5'**					1.51, *s*

^1^ DMSO-d_6_, ^2^ CDCl_3_.

**Table 2:** ^13^C-NMR (125 MHz) spectral data of xanthones **1**–**5**.

Position	Compound 1 ^1^	Compound 2 ^2^	Compound 3 ^2^	Compound 4 ^2^	Compound 5 ^2^
**1**	159.9	159.7	157.9	157.8	160.44
**2**	109.9	111.5	109.6	104.4	108.4
**3**	162.3	163.5	161.5	159.8	161.6
**4**	92.3	88.8	86.7	94.0	93.4
**4a**	154.5	154.4	153.4	156.1	155.3
**5**	101.8	101.5	96.3	101.6	115.2
**6**	156.6	155.6	156.1	154.5	151.0
**7**	143.4	142.5	142.1	142.7	135.8
**8**	136.3	137.0	135.2	136.9	136.5
**8a**	112.2	112.3	112.9	112.1	
**9**	181.3	181.9	180.0	181.8	182.9
**9a**	103.6	103.8	102.0	103.6	117.2
**10a**	154.1	155.2	153.4	155.6	
**11**	21.3	21.3	20.4	115.6	21.4
**12**	122.4	122.3	121.6	126.9	121.4
**13**	130.3	132.0	129.6	77.8	132.6
**14**	25.5	25.8	25.8	28.3	25.8
**15**	17.9	18.2	16.3	25.6?	17.9
**16**	25.6	31.2	24.1	26.5	22.6
**17**	123.7	123.2	120.5	123.1	121.0
**18**	130.3	131.7	129.7	131.8	131.3
**19**	17.7	17.8	15.8	18.1	17.9
**20**	25.7	26.7	24.1	?	25.8
**3-OMe**	−	55.8	54.9		
**6-OMe**	−	−	53.9		
**7-OMe**	60.1	62.0	60.9		
**8-OMe**				7-OMe	
**1'**					121.0
**2'**					131.3
**3'**					77.1
**4'**					27.4
**5'**					27.4

^1^ DMSO-d_6_, ^2^ CDCl_3_.

### 2.2. In Vitro Antiprotozoal and Antimicrobial Activity

The dichloromethane and ethyl acetate extracts of *G. mangostana* were evaluated in an integrated *in vitro* screen for their antiplasmodial, antileishmanial and antitrypanosomal potential (Table 3). While the ethyl acetate extract showed no antiprotozoal activity at all, a pronounced inhibitory effect (IC_50_) was obtained with the dichloromethane extract against *Plasmodium falciparum* (IC_50_ 2.7 µg/mL) and *Trypanosoma brucei* (IC_50_ 0.5 µg/mL), but only with acceptable selectivity (SI) for *T. brucei* (SI 18.8). Some side activity was also noted against *T. cruzi* and *Leishmania infantum* (IC_50_ 7.6 and 7.5 µg/mL), but with low selectivity.

The major constituent α-mangostin was also checked for antimicrobial potential against *Candida albicans*, *Escherichia coli*, *Pseudomonas aeruginosa*, *Bacillius subtilis*, *Staphylococcus aureus*, *Mycobacterium smegmatis*, *M. cheleneoi*, *M. xenopi* and *M. intracellulare* (Table 4). Although inhibitory activity could be indicated against *B.* *subtilis* and *S. aureus* (MIC 1.6 and 3.2 µg/mL) and the *Mycobacterium* species (MIC 1.5 µg/mL), selectivity was quite low in view of the observed cytotoxicity on MRC-5 cells (IC_50_ 7.5 µM) (Table 3). No activity at all was found against *C. albicans*, *E. coli* and *P. aeruginosa* (IC_50_ >200 µg/mL).

**Table 3:** Antiprotozoal activity of *G. mangostana* extracts and α-mangostin.

Sample	*P. falciparum*		*L. infantum*		*T. cruzi*		*T. brucei*		MRC-5
	IC_50_	SI	IC_50_	SI	IC_50_	SI	IC_50_	SI	IC_50_
Dichloromethane extract	2.7 µg	3.5	7.5 µg	3.5	7.6 µg	1.2	0.5 µg	18.8	9.4 µg
Ethyl acetate extract	40.3 µg	>1.6	>64 µg	1	34.6 µg	1.9	56.4 µg	1.1	>64 µg
α-Mangostin	2.2 µM	3.4	8 µM	<1	8.9 µM	<1	7.9 µM	<1	7.5 µM

**Table 4:** Antimicrobial activity (IC_50_) of α-mangostin.

	*B. subtilis*	*C. albicans*	*E. coli*	*P. aeruginosa*	*S. aureus*	*Mycobacterium*			
						*smegmatis*	*cheleneoi*	*xenopi*	*intracellulare*
MIC (µM)	3.9	>200			7.8	3.7	3.7	3.7	3.7

To the best of our knowledge, no data exist in the literature regarding the antiprotozoal activity and potential significance of *G.* *mangostana* as a source of antitrypanosomal and antiplasmodial compounds. *G. parvifolia* (Miq) has been used as a herbal remedy to treat malaria [21] and α-mangostin was found active against *P. falciparum* with IC_50_ values of 5.1 and 17 µM [22,23]. In our study, α-mangostin was found slightly more potent (IC_50_ 2.2 µM), but also cytotoxic to MRC-5 cells (IC_50_ 7.5 µM), hence suggesting a non-specific inhibition. The latter also explains the observed activity against *L.* *infantum*, *T. brucei* and *T. cruzi*, with IC_50_ values between 8.0 and 9.0 µM (Table 3). Another illustration of non-selectivity are several studies quoting the antimicrobial potential of *G. mangostana* extract [24,25]. However, the observed IC_50_ values may still justify the claimed (topical) uses of *G.* *mangostana* to treat infections in the traditional medicine.

This study clearly illustrates that interpretation of the antiprotozoal and antimicrobial potential of prenylated xanthones proves to be far from easy in view of the low level of selectivity. Available data in literature must be interpreted with great caution, particularly when parallel cytotoxicity data are not available. One route of further research on xanthones could be through structural modification with the sole option to maximize efficacy and reduce toxicity, e.g., non-selectivity.

## 3. Experimental Section

### 3.1. General

The UV and IR spectra were recorded on Hitachi-UV-3200 and JASCO 320-A spectrometers. The ^1^H-, ^13^C-NMR and 2D-NMR spectra were recorded on a Bruker AMX-500 spectrometer with tetramethylsilane (TMS) as internal standard. Chemical shifts are given in ppm (*δ*) relative to tetramethylsilane internal standard and scalar coupling constants (*J*) are reported in Hertz. FAB and HRFABMS (neg. ion mode, matrix: glycerol) were registered on a JEOL JMS-HX110 mass spectrometer. Thin layer chromatography (TLC) was performed on precoated silica gel F254 plates (E. Merck, Darmstadt, Germany); detection was done at 254 nm and by spraying with *p*-anisaldehyde/H_2_SO_4_ reagent. All chemicals were purchased from Sigma Chemical Company (St. Louis, MO, USA).

### 3.2. Plant Material

The fruits of *G. mangostana* Linn. were purchased from a local market at Riyadh city in 2009.

### 3.3. Extraction and Isolation

The air-dried pericarp (500 gm) was extracted by maceration with 70% ethanol (3 × 2 L) at room temperature. After filtration and evaporation of the solvent under vacuum, the combined ethanolic extract (70 gm) was suspended in water (200 mL) and successively partitioned with *n*-hexane (3 × 400 mL), dichloromethane (3 × 400 mL) and ethyl acetate (3 × 400 mL) to deliver the corresponding extracts. Based on pattern of separation and close similarity of compounds on TLC examination for both *n*-hexane and dichloromethane extracts, they were pooled together. The combined fractions were further purified by application onto the top of a silica gel packed column (Merck), eluted with *n*-hexane/ethyl acetate, followed by ethyl acetate/methanol solvent system gradient to give five fractions A–F. Fractions A, B and C were separately purified by chromatotron (Harrison Research, Palo Alto, California, CA, USA) using 5%, 15% and 20% ethyl acetate/*n*-hexane to give compound **3** (12 mg), **4** (8 mg) and **5** (20 mg). Direct crystallization of fractions D and E eluted by 30% and 40% ethyl acetate/*n*-hexane gave compound **2** (10 mg) and **1** (300 mg), while direct crystallization of fraction F eluted by 5% methanol/ethyl acetate gave compound **6** (20 mg).

### 3.4. Spectral Data (Table 1 and Table 2)

*Trihydroxy-7-methoxy-2,8-diprenylxanthone* (α-mangostin) (**1**)*.* Yellow amorphous powder; m.p. 180–182 °C; HREIMS: *m/z* = 410.1729 (calc. for C_24_H_26_O_6_, 410.46). ^1^H-NMR (DMSO, 500 MHz); ^13^C-NMR (*d*-DMSO, 125 MHz): see Table 1 and Table 2.

*1,6-Dihydroxy-3,7-dimethoxy-2,8-diprenylxanthone-* (β-mangostin) (**2**). Pale yellow crystal; m.p. 162–163 °C; HREIMS: *m/z* = 424 calc. for C_25_H_28_O_6_, 424.46. ^1^H-NMR (CDCl3, 500 MHz); ^13^C-NMR (CDCl_3_, 125 MHz): see Table 1 and Table 2.

*1-Hydroxy-3,6,7-trimethoxy-2,8-bis(3-methylbut-2-enyl) xanthone* (**3**). Pale yellow gum; m.p. 152–154 °C; HREIMS: *m/z* = 438.5128 (calc. for C_26_H_30_O_7_, 438.5128). ^1^H NMR (CDCl3, 500 MHz); ^13^C-NMR (CDCl_3_, 125 MHz): see Table 1 and Table 2.

*9-Hydroxycalabaxanthone* (**4**). Bright yellow needles; m.p. 152–154 °C; HREIMS: *m/z* = 408.1572 (calc. for C_24_H_24_O_6_, 408.45). ^1^H-NMR (CDCl_3_, 500 MHz); ^13^C-NMR (CDCl_3_, 125 MHz): see Table 1 and Table 2.

*1,3,6-Trihydroxy-2,5-diprenyl-6',6'-dimethylpyrano(2',3':7,8) xanthone* (tovophyllin A) (**5**). Yellow needles; m.p. 218–220 °C; HREIMS: *m/z* = 462.541 (calc. for C_28_H_30_O_6_, 462.2042). ^1^H-NMR (CDCl_3_, 500 MHz); ^13^C-NMR (CDCl_3_, 125 MHz): see Table 1 and Table 2.

*3,5,7,3',4'-Pentahydroxyflavan* (Catechin) (**6**). Cryst.; m.p. 175–177 °C; HREIMS: *m/z* = 290.0790 (calc. for C_15_H_14_O_6_, 290.272). ^1^H-NMR (CDCl3, 500 MHz): 2.74 (*dd*, *J* = 16.5, 4.5 Hz, H-4a) 2.86 (*dd*, *J* = 16.5, 4.5 Hz, H-4b), 4.19 (*m*, H-3), 4.83 (br*s*, H-2), 5.94 (*d*, *J* = 1.5 Hz, H-6), 5.99 (*d*, *J* = 1.5 Hz, H-8), 6.78 (*dd*, *J* = 8.0, 1.0 Hz, H-6'), 6.81 (*d*, *J* = 8.0 Hz, H-5'),7.00 (*d*, *J* = 1.5 Hz, H-2'); ^13^C-NMR (CD3OD, 125 MHz): 80.2 (C-2), 67.5 (C-3), 29.5 (C-4), 157.7 (C-5), 96.5 (C-6), 158.0 (C-7), 96.0 (C-8), 157.4 (C-9), 100.2 (C-10), 132.3 (C-1'), 115.4 (C-2'), 146.0 (C-3'), 145.8 (C-4'), 116.0 (C-5'), 119.5 (C-6').

### 3.5. Reference Drugs

For the different tests, appropriate reference drugs were used as positive control: tamoxifen for MRC-5, chloroquine for *P. falciparum*, miltefosine for *L. infantum*, benznidazole for *T. cruzi* and suramin for *T. brucei*. All reference drugs were either obtained from the fine chemical supplier Sigma-Aldrich (Taufkirchen, Germany; tamoxifen, suramin) or from WHO-TDR (Geneva, Switzerland; chloroquine, miltefosine, benznidazole).

### 3.6. Biological Assays

The integrated panel of microbial screens and standard screening methodologies were adopted as previously described [26]. All assays were performed in triplicate at the Laboratory of Microbiology, Parasitology and Hygiene at the University of Antwerp (Antwerp, Belgium). Extracts were tested at five concentrations (64, 16, 4, 1 and 0.25 μg/mL) to establish a full dose-titration and determination of the IC_50_ (inhibitory concentration 50%). The final in-test concentration of DMSO did not exceed 0.5%, which is known not to interfere with the different assays [26]. The selectivity of activity was assessed by simultaneous cytotoxicity evaluation on the MRC-5 fibroblast cell line. The criterion for activity was an IC_50_ <10 μg/mL and a selectivity index (SI) of >4.

#### 3.6.1. Antiplasmodial Activity

Chloroquine-resistant *P. falciparum* K 1-strain was cultured in human erythrocytes O^+^ at 37 °C under a low oxygen atmosphere (3% O_2_, 4% CO_2_, and 93% N_2_) in RPMI-1640, supplemented with 10% human serum. Infected human red blood cells (200 μL, 1% parasitaemia, 2% haematocrit) were added to each well and incubated for 72 h. After incubation, test plates were frozen at −20 °C. Parasite multiplication was measured using the Malstat assay, a colorimetric method based on the reduction of 3-acetyl pyridine adenine dinucleotide (APAD) by parasite-specific lactate-dehydrogenase (pLDH) [26,27].

#### 3.6.2. Antileishmanial Activity

*L. infantum* MHOM/MA (BE)/67 amastigotes were collected from the spleen of an infected donor hamster and used to infect primary peritoneal mouse macrophages. To determine *in vitro* antileishmanial activity, 3 × 10^4^ macrophages were seeded in each well of a 96-well plate. After 2 days outgrowth, 5 × 10^5^ amastigotes/well, were added and incubated for 2 h at 37 °C. Pre-diluted plant extracts were subsequently added and the plates were further incubated for 5 days at 37 °C and 5% CO_2_. Parasite burdens (mean number of amastigotes/macrophage) were microscopically assessed on 500 cells after Giemsa staining of the testplates, and expressed as a percentage of the blank controls without plant extract.

#### 3.6.3. Antitrypanosomal Activity

*Trypanosoma brucei* Squib-427 strain (suramin-sensitive) was cultured at 37 °C and 5% CO_2_ in Hirumi-9 medium, supplemented with 10% fetal calf serum (FCS) [28]. About 1.5 × 10^4^ trypomastigotes/well were added to each well and parasite growth was assessed after 72 h at 37 °C by adding resazurin [29]. For Chagas disease, *T. cruzi* Tulahuen CL2 (benznidazole-sensitive, LacZ-reporter strain) [30] was maintained on MRC-5 cells in minimal essential medium (MEM) supplemented with 20 mM L-glutamine, 16.5 mM sodium hydrogen carbonate and 5% FCS. In the assay, 4 × 10^3^ MRC-5 cells and 4 × 10^4^ parasites were added to each well. After incubation at 37 °C for 7 days, parasite growth was assessed by adding the substrate chlorophenol red α-D-galactopyranoside. The color reaction was read at 540 nm after 4 h and absorbance values were expressed as a percentage of the blank controls.

#### 3.6.4. Antimicrobial Activity

Samples were tested for antimicrobial activity according to the Clinical Laboratory Standard Institution using American type of Culture Collection (ATCC) standard [31].

#### 3.6.5. Cytotoxicity Assay

MRC-5 SV_2_ cells were cultivated in MEM, supplemented with L-glutamine (20 mM), 16.5 mM sodium hydrogen carbonate and 5% FCS. For the assay, 10^4^ MRC-5 cells/well were seeded onto the test plates containing the pre-diluted sample and incubated at 37 °C and 5% CO_2_ for 72 h. Cell viability was assessed fluorimetrically after 4 h of addition of resazurin. Fluorescence was measured (excitation 550 nm, emission 590 nm) and the results were expressed as % reduction in cell viability compared to control [26].

## 4. Conclusions

Interpretation of the antiprotozoal and antimicrobial potential of prenylated xanthones proves to non-conclusive in view of the low level of selectivity. One route of further research on this subject could be through structural modification with the sole option to maximize efficacy and avoid non-selectivity.

## References

[1] Chen L.G., Yang L.L., Wang C.C. (2008). Anti-inflammatory activity of Mangostins from *Garcinia mangostana*. Food Chem. Toxicol..

[2] Chin Y.W., Kinghorn A.D. (2008). Structural characterization, biological effects, and synthetic studies on xanthones from mangosteen (*Garcinia mangostana*), a popular botanical dietary supplement. Min. Rev. Org. Chem..

[3] Balasubramanian K., Rajagopalan K. (1988). Novel xanthones from *Garcinia mangostana*, structures of BR-xanthone-A and BR-xanthone-B. Phytochemistry.

[4] Pedraza-Chaverri J., Cárdenas-Rodríguez N., Orozco-Ibarra M., Pérez-Rojas J.M. (2008). Medicinal properties of mangosteen (*Garcinia mangostana*). Food Chem. Toxicol..

[5] Moongkarndi P., Kosem N., Kaslungka S., Luanratana O., Pongpan N., Neungton N. (2004). Antiproliferation, antioxidation and induction of apoptosis by *Garcinia mangostana* (Mangosteen) on SKBR3 human breast cancer cell line. J. Ethnopharmacol..

[6] Kaomongkolgit R., Chaisomboon N., Pavasant P. (2011). Apoptotic effect of alpha-mangostin on head and neck squamous carcinoma cells. Arch. Oral. Biol..

[7] Yua L., Zhao M., Yang B., Bai W. (2009). Immunomodulatory and anticancer activities of phenolics from *Garcinia mangostana* fruit pericarp. Food Chem..

[8] Ji X., Avula B., Khan I.A. (2007). Quantitative and qualitative determination of six xanthones in *Garcinia mangostana* L. by LC-PDA and LC-ESI-MS. J. Pharm. Biomed. Anal..

[9] Peres V., Nagem T.J., Faustino de Oliveira F. (2000). Tetraoxygenated naturally occurring xantones. Phytochemistry.

[10] Williams R.B., Hoch J., Glass T.E., Evans R., Miller J.S., Wisse J.H., Kingston D.G.I. (2003). A novel cytotoxic guttiferone analogue from *Garcinia macrophylla* from the Surinam rainforest. Planta Med..

[11] Shan T., Ma Q., Guo K., Liu J., Li W., Wang F., Wu E. (2011). Xanthones from mangosteen extracts as natural chemopreventive agents: Potential anticancer drugs. Curr. Mol. Med..

[12] Bennett G.J., Lee H.H. (1989). Xanthones from Guttiferae. Phytochemistry.

[13] Sen A.K., Sarkar K.K., Mazumder P.C., Banerji N., Uusvuori R., Hase T.A. (1982). The structure of garcinones A, B and C: Three new xanthones from *Garcinia mangostana*. Phytochemistry.

[14] Ghazali S.I.S., Lian G.E., Abd Ghani K.D. (2010). Chemical constituent from roots of *Garcinia mangostana* (Linn.). Int. J. Chem..

[15] Nilar, Harrison L.J. (2002). Xanthones from the heartwood of *Garcinia mangostana*. Phytochemistry.

[16] Sen A.K., Sarkar K.K., Majumder P.C., Banerji N. (1981). Minor xanthones of *Garcinia mangostana*. Phytochemistry.

[17] Trisuwan K., Ritthiwigrom T. (2012). Benzophenone and xanthone derivatives from the inflorescences of *Garcinia cowa*. Arch. Pharm. Res..

[18] Antônio A.A.L., De Oliveira W.G., Taveira Neiva R.M. (1975). Xanthones from *Tovomita pyrifolium*. Phytochemistry.

[19] Bennett G.J., Harrison L.J., Sia G.L., Sim K.Y. (1993). Triterpenoids, tocotrienols and xanthones from the bark of *Cratoxylum cochinchinense*. Phytochemistry.

[20] Ejele A.E., Iwu I.C., Enenebeaku C.K., Ukiwe L.N., Okolue B.N. (2012). Bioassay-guided isolation, purification and partial characterization of antimicrobial compound from basic metabolite of *Garcinia Kola*. J. Emerg. Trends Eng. Appl. Sci..

[21] Faizatun S., Rahayu L. (2009). HPLC analysis and pharmacokinetic study of mangostin after orally administration in rats. Int. J. Pharm. Bio. Sci..

[22] Riscoe M., Kelly J.X., Winter R. (2005). Xanthones as antimalarial agents: Discovery, mode of action, and optimization. Curr. Med. Chem..

[23] Mahabusarakam W., Kuaha K., Wilairat P., Taylor W.C. (2006). Prenylated xanthones as potential antiplasmodial substances. Planta Med..

[24] Priya V., Jainu M., Mohan S.K., Saraswathi P., Gopan S.C. (2010). Antimicrobial activity of pericarp extract of *Garcinia mangostana* Linn. Int. J. Pharm. Sci. Res..

[25] Chomnawang M.T., Sakagami S.S., Nukoolkarn V.S., Gritsanapan W. (2005). Antimicrobial effects of Thai medicinal plants against acne-inducing bacteria. J. Ethnopharmacol..

[26] Cos P., Vlietinck A.J., Berghe D.V., Maes L. (2006). Anti-infective potential of natural products: How to develop a stronger *In vitro* proof-of-concept. J. Ethnopharmacol..

[27] Makler M.T., Ries J.M., Williams J.A., Bancroft J.E., Piper R.C., Hinrichs D.J. (1993). Parasite lactate dehydrogenase as an assay for *Plasmodium falciparum* drug sensitivity. Am. J. Trop. Med. Hyg..

[28] Hirumi H., Hirumi K. (1989). Continuous cultivation of *Trypanosoma brucei* blood stream forms in a medium containing a low concentration of serum protein without feeder cell layers. J. Parasitol..

[29] Raz B., Iten M., Grether-Buhler Y., Kaminsky R., Brun R. (1997). The Alamiar Blue assay to determine drug sensitivity of African trypanosomes (*T. b. rhodesiense, T. b. gambiense*) *in vitro*. Acta Trop..

[30] Buckner F.S., Verlinde C.L., la Flamme A.C., van Voorhis W.C. (1996). Efficient technique for screening drugs for activity against *Trypanosoma cruzi* using parasites expressing beta-galactosidase. Antimicrob. Agents Chemother..

[31] Ferraro M.J. (1997). National committee for Clinical Laboratory Standards. Methods for Dilution Antimicrobial Susceptibility Tests for Bacteria That Grow Aerobically.

